# Treatment of Graves's Disease by Anti-Thyreoid Serum and by X-Rays
1Read at a meeting of the Bristol Medico-Chirurgical Society held at Weston-super-Mare on June 12th, 1907.


**Published:** 1907-09

**Authors:** J. Michell Clarke

**Affiliations:** Professor of Medicine, University College, Bristol; Physician to the Bristol General Hospital.


### TREATMENT OF GRAVES'S DISEASE BY ANTI-THYREOID SERUM AND BY X-RAYS.[1]

BY

J. MICHELL CLARKE, M.A., M.D. Cantab., F.R.C.P.,

*Professor of Medicine, University College, Bristol; Physician to the Bristol General Hospital.*

---

It may be well, before passing to the subject of treatment, to recall briefly the chief theories as to the nature of Graves's disease. Unfortunately the pathology of this affection is still very obscure, and the exact way in which it is produced uncertain.

Undoubtedly many of the most striking symptoms are brought about through the medium of the sympathetic, but there is no evidence that the disease originates in an affection of the sympathetic; and similarly, though the three cardinal symptoms have been produced by experimental lesion of the restiform bodies, and though there is even a stronger point in favour of the theory which would attribute the disease to a lesion of the medulla oblongata, namely that morbid changes are fairly constantly found there, there is no sufficient proof that these lesions cause Graves's disease or are more than secondary ones. The theory that it is due to hypersecretion of the thyroid has perhaps more to support it. Myxœdema is in some respects the antithesis of Graves's disease, and we know that the former depends on an absence of the thyroid secretion. Again, much has been made of the resemblance between the symptoms of Graves's disease and those produced by an overdose of thyroid extract; but though there is a certain analogy between the two conditions, it must be objected that no one has ever yet succeeded in producing the full clinical picture of Graves's disease by administering thyroid extract.

It was on the supposition that the disease depended on a

[1] Read at a meeting of the Bristol Medico-Chirurgical Society held at Weston-super-Mare on June 12th, 1907.

hypersecretion by the thyroid that Möbius advised the use of serum from thyroidectomised goats. He supposed that in the serum of such animals there would accumulate an excess of anti-bodies (to the thyroid), and that these, when ingested by the patient suffering from Graves's disease, would neutralise the excess of thyroid secretion in them.

Numerous observers have published good results obtained from the use of such sera. The serum was at first injected, but was afterwards found to be equally efficacious by the mouth. Good results have also been alleged from the use of Rodagen—the milk of thyroidectomised goats—given in the form of tablets.

I give my experience of the use of these remedies below. Of the new serum prepared by Rogers and Beebe I have had no experience; their method is also founded on the thyroid hyper-secretion theory of Graves's disease, but based on a different principle. They first endeavoured to find out how the thyroid secretion in this disease differs from that in health, obviously a most important point, and one not satisfactorily settled. As a result of an extensive investigation, Oswald says that the only difference between the two is that the thyroid secretion in Graves's disease contains less iodine. The objects at which Rogers and Beebe aimed are (1) to neutralise toxic substances, and (2) to stop their production in quantity. From the nucleo-proteids of certain organs they prepared a serum which had a specific cytolytic effect on these organs, by inoculating rabbits (1) with nucleo-proteids and thyroglobulins from the thyroid glands of fatal cases of Graves's disease, and (2) with the same substances from normal thyroids. After a definite number of injections, the activity of the serum of these animals was determined by an agglutinin test and injections made into patients with Graves's disease. They claim that of the cases thus treated 11 were cured, 43 improved, and 15 were unaffected.

I have treated three well-marked cases of exophthalmic goître with Rodagen; the patients improved—but I think not more than most cases improve by rest in hospital—and I was not able to trace any distinct effect from the remedy. I give the details of one case.

**Case 1.**—Rosina H., æt. 26. Duration of thyroid swelling, tachycardia, and tremor with general weakness, &c., nine years. Exophthalmos first appeared eighteen months previous to admission. Her condition has varied from time to time, being sometimes better, sometimes worse; after each of her two confinements she was decidedly worse. On admission she had marked exophthalmos, pulsating goître and tremor; pulse-rate 140. The heart was slightly enlarged, and there was a systolic apex murmur. For the first week, under ordinary treatment by rest, phosphate of soda and glycero-phosphates, the patient felt better, and the pulse-rate was from 118—130. From November 29th to December 12th Rodagen was given in half-drachm doses three times a day. The pulse varied from 118—130; the neck measurement remained the same. The patient declared herself to be feeling decidedly improved, and there was obviously a general improvement in her condition. From December 12th to 20th Rodagen was stopped without making any apparent difference, and was given again from December 20th to January 23rd. The pulse-rate remained the same, proptosis and thyroid enlargement unaltered, heart's action less excited. There was less tremor, and she said the palpitation was less. As a result of the seven weeks' treatment she gained 8 lb. in weight. Subsequently this patient was treated with Möbius's antithyreoid serum, in a dose of 5 cc. daily for about three weeks. The patient continued to show improvement on her state on admission. The tremor became decidedly less, and the pulse-rate 112—120. No other objective sign of improvement was noted, but she stated that she felt very much better.

The following cases were treated with antithyreoid serum (Möbius):—

**Case 2.**—Louisa B., æt. 36, married. Never good health. Duration of illness, two years. Goître, palpitation, exophthalmos, tremor. No cardiac enlargement, no apex murmur. Dyspepsia and vomiting, throbbing of vessels. Admitted March 15th, 1907. Pulse-rate 140 on admission; this with rest dropped to 106 in six days. Neck measured thirteen inches in circumference. March 21st: Antithyreoid serum 5 cc. every other day, and bismuth mixture for indigestion. On April 3rd condition much the same, but pulse-rate 96. She said her neck felt smaller, and it was one inch less on measurement. April 9th, pulse 120. As she said she did not feel so well, and that the heart beat faster after taking the serum, the pulse-rate was taken every hour for eight hours after the dose. It was found that no essential difference could be made out in the pulse-rate after the dose from that on the days that she did not take the serum. The only change was, that on the average the pulse-rate on four occasions was about 10 beats to the minute slower for some hours after the serum, viz. 112—114 instead of 124. On April 17th pulse-rate was 112, perspirations were less profuse, tremors same, general condition was improved. Five cc. antithyreoid serum had been taken every other day from March 21st to April 3rd ; 10 cc. from April 3rd to April 18th. The following estimations were made of the nitrogen in the food and urine ; that in the fæces was not determined ; the bowels were moved regularly once a day, and there was no diarrhœa. On April 17th 10 cc. serum were given, and she had been taking it since March 21st. Her weight was 45 kilos. The food taken on this day contained 1,800 calories ; the nitrogen in the food was 147.5 grains, and nitrogen excreted in urine 126.7 (as urea and in purin bodies separately estimated). On a later day, when no serum had been taken for three days, she took a larger amount of food—over 2,300 calories—and the nitrogen ingested and excreted, estimated in the same way, was respectively 176 and 140 grains.

**Case 3.**—Margaret E., æt. 18. This patient was in the Hospital in the autumn of 1906 with hysterical mutism, anorexia nervosa, tachycardia, tremor and exophthalmos. She was profoundly emaciated, weighing 5 st. 10 lb., whilst her height was 5 ft. 10 in. Under Weir-Mitchell treatment, her weight went up to 8 st. 9 lb. She left the Hospital after three months with a pulse of 140. There was then no thyroid swelling, but this appeared shortly afterwards. Readmitted March 28th, 1907, she was well nourished, with marked exophthalmos, pulsating goître, tremor, profuse sweats, and loss of power in the legs. Pulse-rate, 140 ; heart slightly enlarged, with a loud systolic murmur all over the præcordia. She was ordered antithyreoid serum in dose of 5 cc. every other day. After a week this dose was given daily, and then she took 10 cc. every other day for another week, and during the fourth week 10 cc. every day. Duration of treatment, April 1st to 28th inclusive. No appreciable effect was noticed during the time the serum was administered. On April 1st, on beginning treatment, pulse-rate was 144 ; on April 7th it was 140 ; on 17th it dropped to 112 ; but on April 24th was 150, and on May 15th was 150 ; on June 5th, 135. The respiration was 30. Sweats remained undiminished ; exophthalmos same. The goître—a large one, circumference of neck being fifteen inches—was unaltered ; tremor perhaps less. On the other hand, she expressed herself as feeling better and stronger, and suffering less from palpitation ; she slept better, and her appetite improved. The quantity of urine averaged 44—45 oz. per diem at the commencement of the serum treatment ; dropped to about 30 oz. per diem at the end of it, and for three weeks afterwards. This patient was paraplegic ; the paraplegia, which presented some of the features of hysterical paraplegia in the absence of muscular wasting, of alteration of the reflexes, of dulling of sensation to pain (pin-prick) and of the muscular sense, did not improve under serum treatment. The patient developed a very large appetite; her weight was 9 stone. On April 17th, 18th, the amount of food taken was as follows:—

| | 17th. | 18th. |
|---|---|---|
| Nitrogen in food .. .. | 204.2 grains. | 197.6 grains. |
| Nitrogen excreted in urine (urea and purin bodies) . | 206.7 ,, | 211.5 ,, |

The nitrogen in the fæces was not estimated; there was one daily action of the bowels, so that apparently there was some loss of nitrogen during the time the serum was taken. On May 5th, about a week after serum had been discontinued:

| | |
|---|---|
| Total nitrogen in food .. .. .. | 223 grains. |
| Nitrogen excreted in urine .. .. | 212 ,, |

**Case 4.**—Gladys J., 19 years. First symptoms: goître, exophthalmos, palpitation, appeared six to seven months previous to admission. On admission there were a large pulsating goître with thrill, a moderate degree of exophthalmos, a marked tremor, and tachycardia. Pulse 132, no enlargement of heart, a systolic pulmonary but no apex murmur, and anæmia. Under six weeks' treatment by rest, constant current to neck, strophanthus, belladonna and iron, little or no improvement took place. Pulse-rate averaged 140. From September 14th to 28th she was given antithyreoid serum 5 cc. every other day, all other treatment being stopped. At the end of this time there was slight improvement, consisting of less pulsation in the thyroid, with a diminution in the circumference of the neck from fourteen to thirteen inches, less tremor, and a pulse-rate of 130—124. In other respects the condition was unchanged, and she left the Hospital at her own request a week later without any very material change in her state.

**Case 5.**—Lily T., æt. 26. Symptoms attributed to a fall in August, 1905, when she struck her head, and was in bad health for some time afterwards. Tachycardia, exophthalmos and goître appeared November, 1905. On admission, April, 1906: pulse 110, no cardiac enlargement or murmur, marked tremor, slight exophthalmos, and small goître, chiefly of right lateral lobe of thyroid. She suffered from constant profuse perspirations. Blood pressure, 120 mm. Hg. From April 5th to 30th, 1906, she was treated by rest in bed, by antithyreoid serum, ʒi, given twice daily; and, as she slept badly, an occasional dose of Veronal at night. This was a mild case. She certainly improved under treatment; the pulse-rate fell to 88—98, the blood pressure to 105 mm. Hg. There was less tremor, less exophthalmos; her general condition improved; she became less nervous, and recovered her sleep. On the other hand, the profuse perspirations were unchanged. She gained a few pounds in weight. After April 30th the antithyreoid serum was stopped, and she was given belladonna, with application of a constant current to the neck. There was no material change in her state, the improvement being maintained, but she varied from day to day, having bad days, in which the palpitation was again severe.

In the following cases treatment was by X-rays :—

Case 5 remained in much the same state until August, 1906. From August 11th to November 10th she was treated by X-rays, attending as an out-patient three to four times a week for that purpose. The X-rays were applied over the thyroid gland for ten minutes at each sitting, thirty-four sittings in all. No distinct effect was observed from this course of treatment, except that there was some diminution in the size of the goître.

**Case 6.**—Ida N., æt. 27, was treated by X-rays. Admitted July 31st, 1906. She had had no previous illness, except rheumatic fever two years ago. The present illness began three years ago with a pulsating goître, palpitation and tachycardia. This attack lasted six weeks, and was relieved by rest at a convalescent home. Symptoms returned March, 1906, and continued until admission. On examination there were a large pulsating goître, marked tremor, slight exophthalmos, pulse-rate 108, pigmentation of upper eyelids, and a systolic murmur at apex. From August 4th to September 19th she was treated by application of X-rays to the thyroid on four days a week, for ten minutes at each sitting. At the end of this time the neck measured about the same, pulse-rate 112, pulsation in vessels same, tremor decidedly less. She had lost 4 lb. in weight. She said herself that she felt better. The X-rays were then omitted for a week without any material change in her condition, except that she gained 2 lb. in weight. Treatment by X-rays was resumed on September 26th, and continued until November 10th ; during this time she was an out-patient. She gained weight slowly but steadily during this period, gaining 6 lb. in all. Her pulse remained at 108, the tremor was still present but less. Sleep was good, and she had a good appetite, but suffered from dyspepsia. On the whole, slight general improvement.

**Case 7.**—Nellie B., æt. 23. Illness stated to have begun six months previously. She had taken thyroid extract for psoriasis. On examination there was left-sided exophthalmos, slight left ptosis, a large pulsating goître, pulse-rate 125—130, marked tremor, profuse sweats and general nervousness. Treatment with X-rays was carried out in the same way as in the other patients, forty-three sittings being given in all between August 10th and

November 3rd, 1906. In this case the pulse-rate fell to an average of 116 in October and to 98—100 in November, the goître became smaller, the tremor and sweats less, and the patient felt generally better.

Of another case treated by the X-rays for six weeks I need not give the details, as although a diminution in the size of the thyroid, and a temporary fall in the pulse-rate occurred, except for the former, there was no permanent alteration in the patient's state as a result of treatment.

There is great difficulty in estimating at its true value any mode of treatment of Graves's disease. The malady is not a fatal one ; in a case of average severity it may not materially shorten life. It is not steadily progressive ; remarkable ameliorations occur in the natural course of the illness, and these remissions may last a long time. Further, most of the symptoms are directly produced by functional disorders of the nervous system, For many of these we have to depend on the statements and on the subjective feelings of the patient ; and even of those which can be objectively observed, some are of such a nature as to be influenced by nervous states. The symptoms generally are easily affected by changes, for good or bad, in the general conditions of life. In hospital in-patient practice the effect of treatment is particularly difficult to judge, for the improved conditions arising from good food, rest, freedom from harassing family cares, well-ventilated wards, and general care to maintain the bodily functions in good order, nearly always result in considerable improvement in the patient's health, and are just those best adapted to counteract the disabilities of the disease.

On looking over notes of old cases treated simply by rest, a full diet, tonics, belladonna, and electricity to the thyroid gland, I find that in nearly every case the patient went out better. This statement must be modified to the extent that no patient left the Hospital cured. Goître, exophthalmos, pulse-rate, tremor, sweats, might all be less, but still remained to some extent. Thus I do not find a pulse-rate below 90. The symptoms most completely relieved were "nervousness," tremor, palpitation or subjective sense of pulsation in vessels, sweatings, and sense of suffocation or difficulty of breathing from enlarged thyroid, and together with this relief a general increase of strength and of well-being.

Compared with the ordinary results of hospital treatment, I did not find any striking advantage from the use of antithyreoid serum, though it must be said that the patients certainly declare themselves as feeling much better, more than they usually do under ordinary treatment. So far as I am aware, they did not know that they were taking a new remedy. There was no especial point to notice, except that in two of the cases, so far as observations went, there was an increase of nitrogenous excretion by the urine, and in one the quantity of urine passed was decreased. The expense of the serum is a decided disadvantage.

With regard to the X-rays, more experience is required; as the patients were out-patients, the treatment by X-rays was the only change from their ordinary condition. In one case, which was anomalous in its alleged mode of origin, their use appeared to be attended with marked and permanent benefit. The others "felt better," and there was a slight diminution in pulse-rate during treatment, which was not, however, permanent. In one respect I think the X-rays may be of advantage, and that is in diminishing the size of the goître; this diminution was permanent in three cases, and with it the patients lost the feeling of suffocation, of which they had occasionally complained. In one patient there were marked fluctuations in weight; but though weight was lost during a first course of X-rays, it was gained during a second. Such fluctuations also occur, with remissions, in the natural course of the disease.

It may be added that in each case the urine was normal, and that, from what is now known of treatment by X-rays, it would probably be inadvisable to use them in any case where the kidneys were not sound.

I am much indebted to my colleague, Dr. W. K. Wills, for the care with which he carried out this part of the treatment.

